# MicroRNA-1182 and let-7a exert synergistic inhibition on invasion, migration and autophagy of cholangiocarcinoma cells through down-regulation of NUAK1

**DOI:** 10.1186/s12935-021-01797-z

**Published:** 2021-03-09

**Authors:** Xin Pan, Gang Wang, Baoming Wang

**Affiliations:** Interventional Department, The Fourth Affiliated Hospital of China Medical University, No. 4, Chongshan East Road, Huanggu District, Shenyang, 110032 Liaoning People’s Republic of China

**Keywords:** MicroRNA-1182, Let-7a, NUAK1, Cholangiocarcinoma, Invasion, Migration, Autophagy

## Abstract

**Background:**

Cholangiocarcinoma (CCA) is the second most common primary liver malignancy worldwide. Several microRNAs (miRNAs) have been implicated as potential tumor suppressors in CCA. This study aims to explore the potential effects of miR-1182 and let-7a on CCA development.

**Methods:**

Bioinformatics analysis was conducted to screen differentially expressed genes in CCA, Western blot analysis detected NUAK1 protein expression and RT-qPCR detected miR-1182, let-7a and NUAK1 expression in CCA tissues and cell lines. Dual luciferase reporter gene assay and RIP were applied to validate the relationship between miR-1182 and NUAK1 as well as between let-7a and NUAK1. Functional experiment was conducted to investigate the role of miR-1182, let-7a and NUAK1 in cell migration, proliferation and autophagy. Then, the CCA cells that received various treatments were implanted to mice to establish animal model, followed by tumor observation and HE staining to evaluate lung metastasis.

**Results:**

CCA tissues and cells were observed to have a high expression of NUAK1 and poor expression of miR-1182 and let-7a. NUAK1 was indicated as a target gene of miR-1182 and let-7a. Importantly, upregulation of either miR-1182 or let-7a induced autophagy, and inhibited cell progression and in vivo tumor growth and lung metastasis; moreover, combined treatment of miR-1182 and let-7a overexpression presented with enhanced inhibitory effect on NUAK1 expression and CCA progression, but such synergistic effect could be reversed by overexpression of NUAK1.

**Conclusion:**

Taken together, the findings suggest the presence of a synergistic antitumor effect of miR-1182 and let-7a on the development of CCA via the down-regulation of NUAK1, providing novel insight into the targeted therapy against CCA.

## Background

As the second-most common primary hepatobiliary malignancy, cholangiocarcinoma (CCA) generally occurs as a result of long-standing inflammation, injury and reparative biliary epithelial cell proliferation [1, 2]. The high mortality of CCA is explained by the extreme difficulty associated with the diagnosis of CCA at an early stage, along with high frequency of recurrence and metastasis and lack of effective chemotherapeutical, surgical or other treatment options [3]. The risk factors of CCA include cirrhosis and viral hepatitis B and C [4]. On the other hand, overexpression of inflammatory genes, cytokines or STAT3, could result in the development of CCA, especially intrahepatic cholangiocarcinoma (ICC). Additionally, strong correlations have been identified between CCA and genetic mutations in patients [5]. Several genes have been considered as potential therapeutic targets in CCA secondary to their independent prognostic values, such as VDR, CYPA, CD147 and HER4 [6, 7]. Nevertheless, the therapeutic and diagnostic potential of these genes requires further large-scale investigations.

As a member of the human adenosine monophosphate-activated protein kinases family, NUAK1 (also known as ARK5, AMPK-related kinase 5) has been observed to have a high expression in multiple human malignancies, and participates in tumor invasion, migration, survival and progression [8, 9]. For example, NUAK1 is highly expressed in non-small cell lung cancer (NSCLC) and knockdown of this gene served as a block in lung metastasis and invasive ability in a xenograft mouse model [8]. Moreover, NUAK1 is a valuable molecular biomarker for gastric cancer progression, since its suppression can prevent cell invasion, lymph node metastasis, pathological stage and histological differentiation [10]. miRNAs are endogenous noncoding RNAs that serve as modulators of gene expression and their dysregulation has been linked with the development of inflammatory-driven carcinogenesis [11]. Numerous studies have emphasized the tumor suppressor or promoter role of miRNAs, which might function in the tumorigenic process of malignant tumors such as CCA [3, 12–14]. In pancreatic cancer, NUAK1 is specifically up-regulated and the down-regulation by miR-96 is capable of impeding the proliferation, migration and invasion of MIA PaCa-2 pancreatic cancer cells [15]. In addition, NUAK1 level has been shown to be significantly increased in ICC tissues and cell lines while miR-145 expression was significantly decreased; moreover, NUAK1 was also identified as a direct target of miR-145 regulation, aiding the prevention of ICC progression [16]. Autophagy may be related to the development of CCA [17]. As autophagy is essential in numerous physiologies, ranging from immune response to neuronal health, its activation is key in the pathogenesis of CCA progression [18]. Pietri Puustinen et al. demonstrated the presence of a close correlation between NUAK1 and autophagy [19], whilst Chen et al. suggested that the inhibition of NUAK1 suppressed autophagy as indicated by the decreased expression of MMP-2, matrix metalloproteinase (MMP)-2 and MMP-9 [8]. However, the interaction between NUAK1 and autophagy remains unclear. These findings led to the hypothesis that there existed a promising correlation between NUAK1 and other miRNAs in CCA. Therefore, the present study focused on exploring the correlations between the expression of miR-1182 and let-7a and CCA cell migration, invasion and autophagy with aims of discovering a promising therapeutic strategy for CCA.

## Materials and methods

### Ethical statement

This study was carried out in strict accordance with the recommendations of the Guide for the Care and Use of Laboratory Animals of the National Institutes of Health. The protocol was approved by the Institutional Animal Care and Use Committee of The Fourth Affiliated Hospital of China Medical University.

### Bioinformatics analysis

Differentially expressed genes (DEGs) were screened using CAA-related gene expression profile downloaded from the Gene Expression Omnibus (GEO) database (https://www.ncbi.nlm.nih.gov/geo/) and R language "limma" package with threshold set as |logFC|> 2 and *p* < 0.05. DEGs were presented in a heat map by using “pheatmap” package. Subsequently, the expression of NUAK1 in the CCA samples collected in The Cancer Genome Atlas (TCGA) was analyzed based on the UALCAN database (http://ualcan.path.uab.edu/index.html). Next, CCA related genes were retrieved in the MalaCards database (http://www.malacards.org/), and their interaction with NUAK1 was further analyzed in the STRING database (https://string-db.org/). Finally, the RNA22 database (https://cm.jefferson.edu/rna22/Precomputed/) and miRGator database (http://mirgator.kobic.re.kr/) were performed to predict the miRNAs that regulated NUAK1. The intersection of the first 80 miRNAs from the result obtained from the above two databases was analyzed using the Venn diagram website (http://bioinformatics.psb.ugent.be/webtools/Venn/).

### Study subjects

A total of 56 patients confirmed as hilar CCA by surgery and pathology from January 2018 to December 2018 in The Fourth Affiliated Hospital of China Medical University were enrolled as study subject, including 34 males and 22 females, aged 35–78 years old. According to tumor nodes metastasis (TNM) stage of Union for International Cancer Control, there are 22 cases of stage I-II and 34 cases of III-IV. According to the degree of differentiation, 20 cases had poor differentiation, 19 cases had moderate differentiation, and 17 cases had high differentiation. Meanwhile, 32 cases of bile duct tissues were obtained from patients with hepatic trauma specimen and served as control.

Human normal biliary epithelial cell line HIBEPIC and CCA cell lines (CCC-5, HCC-9810, Huh28) from BeNa Culture Collection (Beijing, China) were detected by mycoplasma and identified by short tandem repeat (STR) genotyping (Additional file 1: Table S1). Cells were cultured in dulbecco’s modified eagle medium (DMEM) medium (QBC939) or a Roswell Park Memorial Institute (RPMI) 1640 medium (HCCC-9810 and Huh28) containing 10% serum at 37 °C in a 5% CO_2_ incubator and were sub-cultured upon the cells reaching 80–90% confluence. A reverse transcription quantitative polymerase chain reaction (RT-qPCR) was applied to screen out the cell line with the lowest expression of miR-1182 and let-7a for subsequent experiments.

### Dual-luciferase reporter gene assay

The bioinformatics website (http://microRNA.org) identified NUAK1 as the target gene of miR-1182 and let-7a, after which their binding relationship was determined using dual-luciferase reporter gene assay. Wild type (WT) and mutated (MUT) NUAK1 3′UTR gene fragment containing miR-1182 or let-7a binding target was synthesized. The synthesized fragments were cloned into a pMIR-REPORT plasmid (Guangzhou RiboBio Co., Ltd., Guangdong, China) with application of restriction enzyme. Then the plasmids were co-transfected with miR-1182 mimic, let-7a mimic, or mimic NC into HCCC-9810 and Huh28 cells. Before transfection, HCCC-9810 and Huh28 cells were seeded onto 6-well plates at a density of 2 × 10^5^ cells/well and incubation was carried out in an incubator at 37 °C and 5% CO_2_ overnight, followed by transfection. Then 4 μg of the target plasmid and 2 ug of miRNA with 250 μL serum-free Opti-MEM (Gibco, Grand Island, NY, USA) was mixed in a 1.5 EP tube and 10 μL Lipofectamine 2000 with 250 μL serum-free Opti-MEM (Gibco, Grand Island, NY, USA) medium mixed in another tube for 5 min. After being allowed to stand for 20 min, the mixture was added to the plate followed by incubation with cells in the incubator at 37℃ and 5% CO_2_ for 5–6 h. Then cells were transferred to complete medium containing 10% fetal bovine serum (FBS) and underwent 48-h incubation. Next, luciferase activity was detected using Genecopoeia’s Dual Luciferase Assay Kit (D0010, Beijing Solarbio Science & Technology Co., Ltd.). Fluorescence intensity was measured using a Promega Glomax 20/20 luminometer fluorescence detector (E5311, Shaanxi Zhongmei Biotechnology Co., Ltd., Xian, China).

### Cell transfection

Cells in logarithmic growth period were seeded onto 6-well plates (3 × 10^5^ cells/well) and when cell confluence reached 80%, cells were transfected with miR-1182 mimic, let-7a mimic, miR-negative control (NC), miR-1182 mimic + let-7a mimic, pcDNA1, overexpression (oe)-NUAK1, and miR-1182 mimic + let-7a mimic + oe-NC using Lipofectamine 2000 (Invitrogen, New York, California, USA). Afterwards, 4 μg of the target plasmid or 2 ug miRNA and 10 μL Lipofectamine 2000 were diluted by 250 μL serum free Opti-MEM (Gibco), placed for 5 min and mixed, with the mixture added to the plate well. The cells were cultured in a 37 °C, 5% CO_2_ incubator for 6 h and transferred to complete medium for 48 h.

### RNA isolation and quantification

Total RNA was extracted from tumor tissues with the use of miRNeasy Mini Kit (217,004, Qiagen, Hilden, Germany). Then the total RNA was reversely transcribed into cDNA (10 μL) using the Primescript™ RT reagent Kit (RRO36A, Takara Biotechnology Ltd., Dalian, Liaoning Province, China). All the samples were performed using a SYBR® Premix Ex Taq™ II reagent kit (RR820A, Takara) on an ABI7500 real-time quantitative PCR system (ABI Company, Oyster Bay, NY, USA) with a reaction system (50 μL) consisting of SYBR® Premix Ex TaqTM II (2 ×) 25 μL, PCR upstream primer 2 μL, PCR downstream primer 2 μL, ROX Reference Dye (50 ×) 1 μL, cDNA template 4 μL, and ddH_2_O 16 μL. The primers were all synthesized by Takara and are listed in Table 1. The fold changes were calculated using the relative quantification (2^−ΔΔCt^ method) with 2 μg RNA as template and Glyceraldehyde-3-phosphate dehydrogenase (GAPDH) or U6 as internal reference.

**Table 1:** Primer sequences for RT-qPCR

Gene	Sequence (5′-3′)
miR-1182	F: GGGGAGGGTCTTGGGAGGGA
	R: GTGCAGGGTCCGAGGT
miR-let-7a	F: TGAGGTAGTAGGTTGTGTGGTT
	R: GTGCAGGGTCCGAGGT
NUAK1	F: GAAGTTATGCTTTATTCAC
	R: CATCCTCTGATTCTAGGTG
MMP-2	F: AGTTTCCATTCCGCTTCCAG
	R: CGGTGGTAGTCCTCAGTGGT
MMP-9	F: ACTACTGTGCCTTTGAGTCC
	R: AGAATCGCCAGTACTTCCCA
Beclin1	F: ATGCAGGTGAGCTTCGTGTG
	R: CTGGGCTGTGCTAAGTAATGCA
LC3	F; AATCCCGGTGATCATCGAGC
	R: GCCGGATGATCTTGACCAAC
U6	F: ATTGGAACGATACAGAGAAGATT
	R: GGAACGCTTCACGAATTTG
GAPDH	F: GGGAAACTGTGGCGTGAT
	R: GAGTGGGTGTCGCTGTTGA

*RT-qPCR* reverse transcription quantitative polymerase chain reaction, *MiR-1182* microRNA-1182, *miR-let-7a* microRNA-let-7a, *NUAK1* Novel (nua) kinase family 1, *MMP-2* matrix metalloproteinase-2, *MMP-9* matrix metalloproteinase-9, *LC3* light chain 3, *GAPDH* glyceraldehyde-3-phosphate dehydrogenase

### Western blot analysis

Cells at logarithmic growth phase were lysed. Afterwards, the cell lysates were separated by 10% sodium dodecyl sulfate–polyacrylamide gel electrophoresis (SDS-PAGE) and transferred onto a polyvinylidene fluoride membrane. The membranes were blocked with 5% skim milk for 1 h at room temperature and incubated with diluted primary antibodies purchased from Abcam Inc.(Cambridge, UK): rabbit polyclonal antibody to NUAK1 (1: 500, ab71814), rabbit polyclonal antibody to matrix metallopeptidase 2 (MMP-2, 2.0 μg/mL, ab37150), rabbit polyclonal antibody to MMP-9 (1.0 µg/mL, ab73734), rabbit polyclonal antibody to light chain 3 beta (LC3B) (1.0 μg/mL, ab51520), and rabbit monoclonal antibody to Beclin1 (1: 2000, ab207612). This was followed by incubation with the horseradish peroxidase (HRP)-conjugated goat anti-mouse immunoglobulin G (IgG) secondary antibody (1: 100; HA1003, Shanghai Yanhui Biotechnology Co., Ltd., Shanghai, China) for 1 h. Finally, the enhanced chemiluminescence reagent (ECL808-25, Biomiga, San Diego, CA, USA) was used to visualize the results by the X-ray film (36209ES01, QcbioSicence & Technology Co., Ltd., Shanghai, China), with GAPDH serving as an internal control.

### RNA binding protein immunoprecipitation (RIP)

RIP kit (Millipore, USA) was used to detect the binding potential of NUAK1 mRNA and miR-1182 or let-7a. Cells were lysed with an equal volume of RIPA lysate (P0013B, Biyuntian, Shanghai, China) in an ice bath for 5 min, followed by centrifugation at 14,000 rpm for 10 min at 4 °C, with the supernatant obtained. Part of the cell extract was used as the input, while the remainder was incubated with antibodies for co-precipitation. For each co-precipitation reaction system, 50 µL magnetic beads were resuspended in 100 µL RIP Wash Buffer and incubated with 5 µg antibody against NUAK1 (1 µg/ml, ab23738) or IgG (1:100, ab172730). After washing, the magnetic bead-antibody complex was re-suspended in 900 µL RIP Wash Buffer, and interacted with 100 µL cell extract at 4℃ overnight. The magnetic bead-antibody complex and Input were digested with proteinase K, from which RNA was extracted for subsequent PCR detection. Argonaute2 (Ago2) is the key factor of Ago2 RIP-seq, and Ago2 is also a core component and effector protein of miRISC silencing complex (miRNA-induced silencing complex), which participates in the gene silencing effect mediated by miRNA and siRNA [20, 21]. Therefore, detecting the binding of miR-1182, let-7a, NUAK1 and AGO2 protein can reflect whether miR-1182, let-7a, and NUAK1 belong to the same RNA-induced silencing complex (RISC), and indirectly indicate the binding between miR-1182, let-7a and NUAK1 mRNA.

### Cell counting kit-8 (CCK-8) assay

After transfection for 48 h, the cells were prepared into single cell suspension and suspension was seeded in a 96-well plate (1 × 10^4^/mL) with sex duplicated wells set and incubated in an incubator at 37℃ for 24 h. Next, the plate was added with CCK-8 solution (Hyclone, South Logan, UT, USA) and underwent additional incubation for 2 h. The absorbance (A) value was measured at 450 nm with a microplate reader.

### Scratch test

After 48 h of cell transfection, cells were seeded in 6-well plates at a density of 5 × 10^5^ cells/well. When cells reached 90% confluence, the scratches were made with a sterile pipette tip. Then the floating cells were washed off with PBS, after which serum-free medium was added for further 0.5–1 h culture to promote cell recovery. With the recovery time used as the start, cells were photographed at 0 h and 24 h respectively, and the cell migration distance was measured using the Image-Pro Plus Analysis software (Media Cybernetics, Inc., Washington Street, Boston, MD, USA).

### Transwell assay

The cell invasion was evaluated by Matrigel invasion assay. 50 mg/L Matrigel (corning, USA) was diluted in 0.5% FBS in DMEM medium at a ratio 1:6. Then, the Matrigel gel (60 µL) was added to upper chamber of the bottom membrane of the Transwell chamber using pipette, followed by rinsing to aspirate the excess gel. Then the chamber was placed in the incubator for 4 h. After 48 h of cell transfection, cell suspension was prepared through cell digestion. Then the suspension was seeded to apical chamber (1 × 10^3^ cells/well) and incubation was carried out in a serum-free medium for 24 h at 37 °C and the basolateral chamber was added with a medium containing 10% FBS. The invasive cells on the lower side of the filter were fixed with 5% glutaraldehyde, stained with 0.1% crystal violet, and counted under a microscope from 5 random fields.

### Monodansylcadaverine (MDC) staining

The prepared cell suspension was added into a 6-well plate (1 × 10^5^ cells/well) containing pre-treated sterile coverslips and cultured for 24 h at 37 °C in a 5% CO_2_ incubator. Then the cells were stained with MDC dye (2 μL/well) for 20 min, and fixed in 2 mL 4% paraformaldehyde for 15 min. After removal of paraformaldehyde, the cells were placed in the center of the coverslip (added with the prepared glycerol). Finally, the coverslip was observed under a fluorescence microscope.

### Immunohistochemistry (IHC)

The CCA tissue sample was fixed with 4% paraformaldehyde, embedded and sliced to Sections (5 µm) and the sections were routinely dewaxed and subjected to antigen retrieval by the application of heat. The sections were washed with PBS and blocked in 5% goat serum for 15 min. Then the sections were incubated with primary antibody rabbit polyclonal antibody NUAK1 (1:100, ab203591, Abcam, UK) overnight at 4 °C and biotinylated goat anti rabbit secondary antibody (1:1000, ab6720, Abcam, UK) for 1 h at room temperature. The sections were incubated horseradish peroxidase-conjugated solution for 15 min, washed twice with 0.01 mol/L (pH = 7.4) PBS and developed with 3,3-diaminobenzidine tetrahydrochloride (DAB) solution (Beijing Zhongshan Jinqiao Biotechnology Co., Ltd.) for 3–5 min. Then the samples were counter-stained with hematoxylin for 1–3 min, dehydrated, cleared and sealed neutral gum mount. The sections were observed and photographed under a Primo Star digital microscope (purchased from McAudi Industrial Group Co., Ltd., Guangzhou, China) through 5 random high-power fields. Positive cells (yellow) were counted and the ratio was calculated: Protein positive rate = number of positive cases/total number of cases × 100%.

### Xenograft model of CCA and pulmonary metastasis tumor model

CCA cells were infected with lentivirus expressing miR-1182 agomire, agomir-NC, miR-1et-7a agomir, and miR-1182 agomir + let-7a agomir, respectively. Stably transfected cells (1 × 10^6^; 200 μL) were then inoculated at their right hind leg back of 32 nude mice (n = 8, each group), under anesthesia with 3% pentobarbital sodium. Then the mice were observed once every 7 d to record the length and width of the tumor. The tumor volume was measured as follows: Tumor volume = (length × width)^2^/2. At the 35th day, the mice were euthanatized to collect the tumors (3 tumor samples in each group). The tumors were weighed and recorded. At the same time, the lung tissues of nude mice were obtained and fixed in formalin, embedded in paraffin and cut into sections. Then the sections were subjected to HE staining and observed under a microscope to detect the tumor formation of CCA in the lung tissues.

### Statistical analysis

All data were analyzed using a Statistic Package for Social Science (SPSS) 21.0 statistical software (IBM Corp. Armonk, NY, USA). The measurement data were described as mean ± standard deviation. Comparisons between two groups in an unpaired design were analyzed using unpaired *t*-test. Comparisons among multiple groups were analyzed using the one-way analysis of variance (ANOVA) and followed by Tukey’s post hoc test. The tumor volume changes were compared with two-way ANOVA and followed by Bonferroni post hoc test. Pearson correlation was used to analyze the correlation between two groups. The enumeration data is expressed as rate and the differences between groups were verified using a chi-square test or Fisher’s exact test. A value of *p* < 0.05 was considered to be statistically significant.

## Results

### NUAK1 is highly expressed in CCA samples

**Fig. 1:**
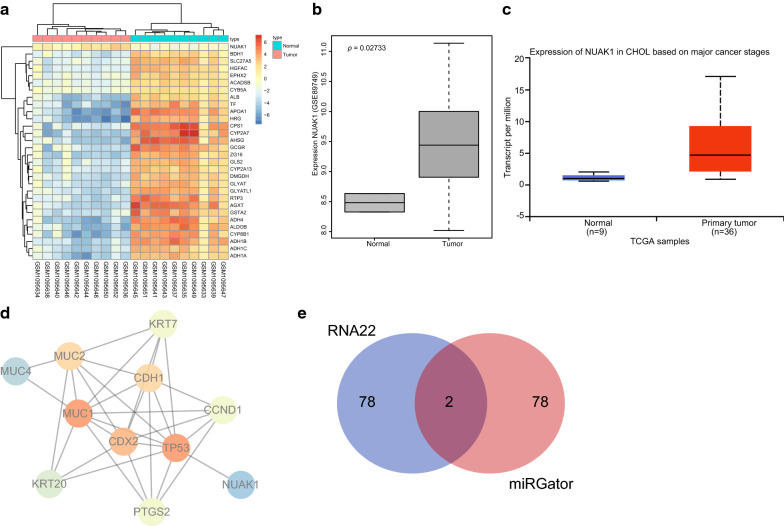
Let-7a and miR-1182 may affect CCA progression via regulating NUAK1. **a** Heat map of DEGs in GSE45001 microarray (the abscissa indicates the sample number, the ordinate indicates the gene name, the upper dendrogram indicates the sample type cluster, and the left dendrogram indicates gene expression cluster, each small square indicates the expression of one gene in one sample, and the histogram in the right upper is color gradation). **b** The expression of NUAK1 gene in the CCA expression microarray GSE89749 (the left box plot represents the expression of NUAK1 in the normal sample, and the right box plot represents the expression of NUAK1 in the CCA sample). **c** NUAK1 gene expression in the TCGA-listed CCA database (the left box plot represents the expression of NUAK1 in the normal sample and the right box plot represents the expression of NUAK1 in the CCA sample). **d** Correlation analysis of NUAK1 and CCA-related genes (each small circle in the Fig. represents one gene; the darker the color, the more central the position of the gene in the network, the line between two genes indicates there is an interaction between them). **e** The prediction result of miRNAs targeting NUAK1 gene, (the left side is the first 80 miRNAs predicted by the RNA22 database, the right side is the first 80 miRNAs predicted by the miRGator database, and the middle part indicates the intersection of the two databases). *CCA* cholangiocarcinoma, *NUAK1* novel (nua) kinase family 1, *DEGs* differentially expressed genes, *TCGA* The Cancer Genome Atlas, *CHOL* cholesterol

Initially, the GEO database was used for the retrieval of CCA related microarray data, after which GSE45001 microarray was obtained. The differences between CCA samples and normal samples were analyzed, and finally 1083 DEGs were obtained. Among them, 404 up-regulated genes were found in CCA along with 679 markedly down-regulated genes. Figure 1a shows an expression heat map of 30 DEGs in the microarray. Among these DEGs, significant up-regulation of NUAK1 gene expression was observed in the CCA samples. Through the GEO database, another CCA-related microarray data GSE89749 was observed, in which the NUAK1 gene was analyzed (Fig. 1b). The results showed significantly increased NUAK1 expression in CCA (*p* < 0.05). The NUAK1 gene expression was further explored in the TCGA-listed CCA database (Fig. 1c), and the results showed that CCA had up-regulated NUAK1 expression. To further confirm the relationship between the NUAK1 gene and CCA, the GeneCards database displayed that one CCA-related gene, tumor protein 53 (TP53), was found to be at the core of the network map, indicating a clear intersection with NUAK1 gene (Fig. 1d). These analyses indicate that NUAK1 is likely to be involved in the development of CCA. To study the upstream regulatory mechanism of NUAK1, the miRNA that can regulate NUAK1 was predicted using the RNA22 and miRGator databases. From intersection of prediction results, there were only two miRNAs, hsa-let-7a and hsa-miR-1182 (Fig. 1e). These analyses suggest that hsa-let-7a and hsa-miR-1182 are highly likely to influence the development of CCA via NUAK1 regulation.

### Differential expression of miR-1182, let-7a and NUAK1 in CCA

**Fig. 2:**
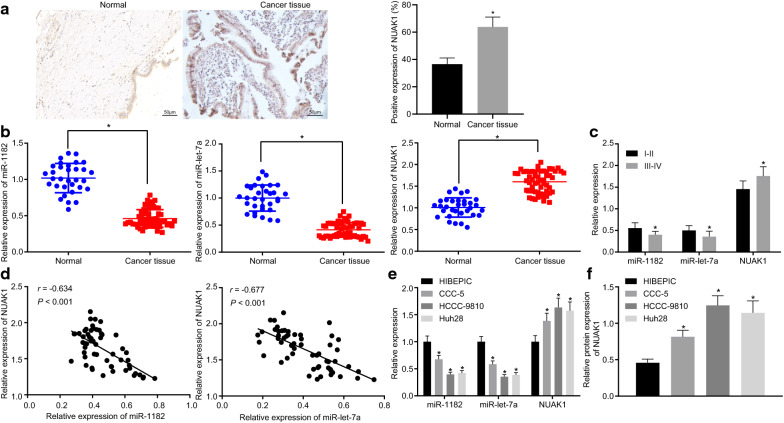
The CCA cell line exhibits decreased expression of miR-1182 and let-7a but increased NUAK1 expression. **a** Representative images of immunohistochemistry of NUAK1 expression in CCA tissue and normal tissues (×200). **b** The expression of miR-1182, let-7a and NUAK1 in CCA tissues (n = 56) and normal tissues (n = 32) determined by RT-qPCR. **c** The expression of miR-1182, let-7a and NUAK1 in different TNM stage (N_I–II_ = 22; N_III–IV_ = 34) determined by RT-qPCR. **d** Pearson analysis of the correlation of the expression of miR-1182, let-7a and NUAK1 in CCA tissues (n = 56). **e** The expression of miR-1182, let-7a and NUAK1 in different cell lines determined by RT-qPCR. **f** The expression of NUAK1 in different cell lines determined by Western blot analysis; ^*^ *p* < 0.05 vs*.* normal cells or HIBEC cells. Statistical data were measurement data and expressed as mean ± standard deviation. Comparisons between two groups were analyzed by independent *t*-test. Comparisons among multiple groups were analyzed using ANOVA and followed by Tukey’s post hoc test. Pearson correlation was used to analyze the correlation between two groups. The experiment was repeated 3 times independently. *ANOVA* analysis of variance

To determine whether miR-1182, let-7a and NUAK1 are differentially expressed in CCA, Immunohistochemistry was first used to identify NUAK1 expression in CCA tissues and controls, the results of which found that NUAK1 was highly expressed in CCA tissues relative to normal tissues (Fig. 2a). RT-qPCR analysis was subsequently performed to determine the expressions of miR-1182, let-7a and NUAK1 in CCA tissues and controls. CCA tissues presented with downregulated expression of miR-1182 and let-7a compared with that of normal tissues, whilst the expression of NUAK1 was up-regulated. Moreover, the aberrant expression of miR-1182, let-7a and NUAK1 was more prominent in the III-IV phase relative to TNM I-II phase (Fig. 2b, c). Pearson correlation analysis showed that there existed a negative correlation between miR-1182 and let-7a and the expression of NUAK in CCA tissues (Fig. 2d).

The relationships between miR-1182, let-7a and clinical pathological features of patients with CCA were further explored. The expression of miR-1182 and let-7a showed no significant difference from the age, sex and tumor size of patients, but their expression was significantly correlated with patient TNM staging, degree of differentiation and lymph node metastasis (LNM) (*p* < 0.05, Table 2). Furthermore, the expression of miR-1182, let-7a and NUAK1 was determined in human normal biliary epithelial cell line HIEPIC and CCA cell lines CCC-5, HCCC-9810, Huh28. Using Western blot and RT-qPCR analyses, the results of which showed decreased expression of miR-1182 and let-7a and increased expression of NUAK1 in CCA cells (Fig. 2e, f).

**Table 2:** The relationship between miR-1182, miR-let-7a and clinicopathological characteristics of patients with CCA

Influence factors	miR-1182			miR-let-7a		
	High	Low	*p* value	HIGH	LOW	*p* value
Gender						
Male						
34	14	20	0.289	17	17	0.74
Female						
22	6	16		10	12	
Age						
≥ 55						
20	7	13	0.934	9	11	0.72
< 55						
36	13	23		18	18	
Tumor size						
≥ 2 cm						
16	6	10	0.86	7	9	0.672
< 2 cm						
40	14	26		20	20	
TNM stage						
I–II						
22	17	5	< 0.001	18	4	< 0.001
III–IV						
34	3	31		9	25	
Differentiation degree						
High						
17	15	2	< 0.001	15	2	< 0.001
Moderate						
19	4	15		10	9	
Low						
20	1	19		2	18	
LNM						
Yes						
27	1	26	< 0.001	3	24	< 0.001
No						
29	19	10		24	5	

The expression of miR-1182 and miR-let-7a was not significantly different from the age, sex and tumor size of patients, but the expression was significantly correlated with patient TNM staging, degree of differentiation and LNM (*p* < 0.001)

*miR-1182* microRNA1182, *miR-let-7a* microRNA-let-7a, *TNM* tumor nodes metastasis, *LNM* lymph node metastasis

### NUAK1 mRNA binds to miR-1182 and let-7a

**Fig. 3:**
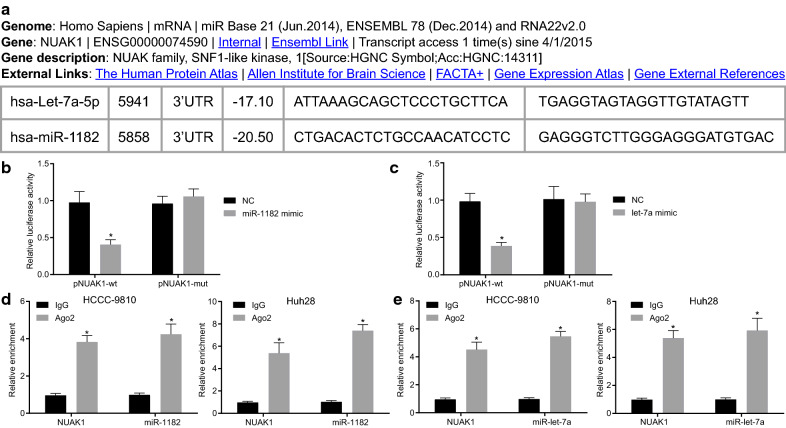
NUAK1 is predicted to be regulated by miR-1182 and let-7a. **a** Prediction of binding sites of miR-1182 and let-7a to NUAK1 mRNA by bioinformatics analysis. **b** Dual-luciferase reporter gene assay of relative luciferase activity of pNUAK1-WT and pNUAK1-MUT upon treatment with let-7a mimic or miR-1182 mimic. **c** RIP experiment of relative enrichment of NUAK1, miR-1182, and let-7a upon addition of IgG or Ago2 in HCCC-9810 and Huh28 cells. **d** RT-qPCR analysis of NUAK1 mRNA expression upon treatment with NC, let-7a mimic or miR-1182 mimic. **e** Western blot analysis of NUAK1 protein expression upon treatment with NC, let-7a mimic or miR-1182 mimic and corresponding quantification. ^*^ *p* < 0.05 vs. the NC group or IgG group. Statistical data were measurement data, described as mean ± standard deviation. Comparisons between two groups were analyzed by independent *t*-test. The experiment was repeated 3 times independently. *NUAK1* novel (nua) kinase family 1, *miR-1182* microRNA-1182, *let-7a* microRNA-let-7a, *NC* negative control

The relationship between NUAK1 and let-7a and miR-1182 is displayed in Fig. 3a. Dual-luciferase reporter gene assay was carried out to verify whether miR-1182 and let-7a can target NUAK1. The results showed a decrease in the luciferase activity of pNUAK1-WT in HCCC-9810 and Huh28 cells upon treatment with miR-1182 mimic and let-7a mimic (*p* < 0.05). However, when the same experiment was conducted with MUT pNUAK1, there was no significant change observed in luciferase activity in the presence of miR-1182 mimic and let-7a mimic (*p* > 0.05). This indicated that NUAK1 mRNA can specifically bind to miR-1182 and let-7a (Fig. 3b). In addition, the binding between miR-1182, let-7a and NUAK1 mRNA was identified with the application of RIP experiments, which revealed that miR-1182 or let-7a resulted in the significant enrichment of NUAK1 (Fig. 3c). The aforementioned findings indicate that miR-1182 and let-7a can bind to NUAK1 mRNA and negatively regulate its expression.

### MiR-1182 and let-7a down-regulates NUAK1 expression in CCA cell line

**Fig. 4:**
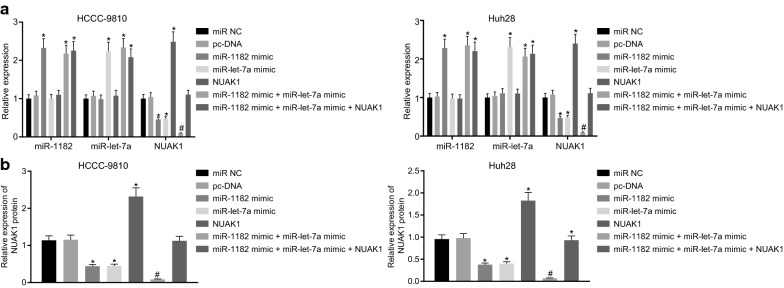
miR-1182 and let-7a down-regulate NUAK1 expression. **a** RT-qPCR analysis of the expression of miR-1182, let-7a and NUAK1 mRNA in HCCC-9810 and Huh28 cells upon treatment with miR NC, pc-DNA, miR-1182 mimic, let-7a mimic, miR-1182 mimic + let-7a mimic, miR-1182 mimic + let-7a mimic + NUAK1. **b** Quantification of NUAK1 protein expression upon treatment with miR-NC, pc-DNA, miR-1182 mimic, let-7a mimic, miR-1182 mimic + let-7a mimic, miR-1182 mimic + let-7a mimic + NUAK1 in HCCC-9810 and Huh28 cells. ^*^ *p* < 0.05 vs. that of cells transfected with miR-NC or pc-DNA, ^#^ *p* < 0.05 vs. that of cells transfected with miR-1182 mimic or let-7a mimic, and & *p* < 0.05 vs. that of cells transfected miR-1182 mimic + let-7a mimic; statistical data were measurement data, described as mean ± standard deviation. Comparisons among multiple groups were analyzed using ANOVA and followed by Tukey’s post hoc test. The experiment was repeated 3 times independently. *GAPDH* glyceraldehyde-3-phosphate dehydrogenase, *RT-qPCR* reverse transcription quantitative polymerase chain reaction

To observe the expression of miR-1182, miR-1et-7a, and NUAK1 in HCCC-9810 and Huh28 cells after transfection, RT-qPCR and Western blot analysis were performed (Fig. 4). Compared with miR-NC and pc-DNA, miR-1182 mimic, let-7a mimic and miR-1182 mimic + let-7a mimic resulted in the significant increase in the expression of miR-1182; similarly, miR-1et-7a mimic, miR-1182 mimic + let-7a mimic, miR-1182 mimic + let-7a mimic + NUAK1 resulted in increased miR-1et-7a expression. Interestingly, NUAK1 expression was increased in cells post overexpression, while the combination of oe-NUAK1, miR-1182-mimic, and miR-1et-7a-mimic restored expression of NUAK1, almost close to the level that was observed upon miR-NC or pc DNA (*p* > 0.05). miR-1182 mimic alone or miR-1et-7a mimic resulted in the reduction of NUAK1 expression, while combined treatment with miR-1182 mimic and let-7a mimic led to further reduction of NUAK1 expression (*p* < 0.05). The aforementioned results indicated that miR-1182 and let-7a overexpression down-regulates NUAK1 levels in HCCC-9810 and Huh28 CCA cells, and NUAK1 expression is restored after NUAK1 overexpression.

### Up-regulated miR-1182 and let-7a inhibit proliferation, migration and invasion of CCA cells

**Fig. 5:**
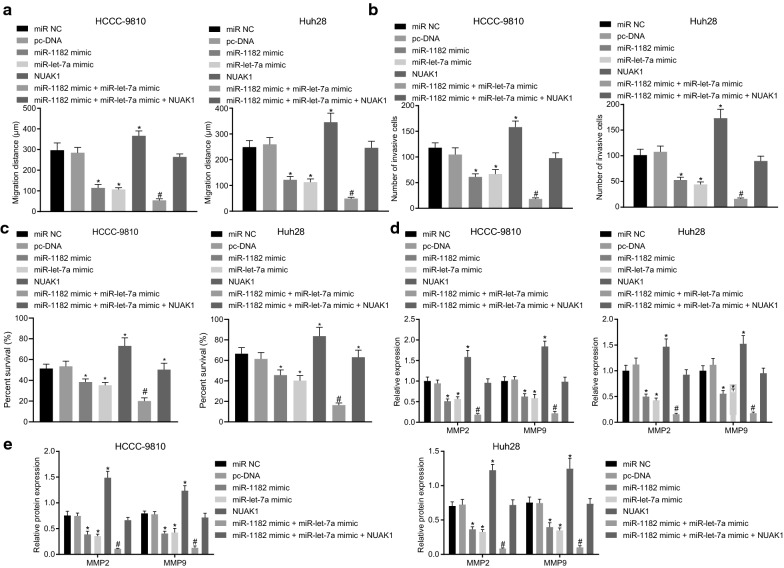
Up-regulated miR-1182 and let-7a disrupt cell invasion and migration. **a** Cell migration distance at 0 h and 24 h using scratch test upon treatment with miR NC, pc-DNA, miR-1182 mimic, let-7a mimic, miR-1182 mimic + let-7a mimic, miR-1182 mimic + let-7a mimic + NUAK1. **b** Cell invasion images using transwell assay upon treatment with miR NC, pc-DNA, miR-1182 mimic, let-7a mimic, miR-1182 mimic + let-7a mimic, miR-1182 mimic + let-7a mimic + NUAK1. **c** Cell viability detected by CCK-8 assay upon treatment with miR NC, pc-DNA, miR-1182 mimic, let-7a mimic, miR-1182 mimic + let-7a mimic, miR-1182 mimic + let-7a mimic + NUAK1. **d** The mRNA expression of MMP2 and MMP9 by RT-qPCR upon treatment with miR NC, pc-DNA, miR-1182 mimic, let-7a mimic, miR-1182 mimic + let-7a mimic, miR-1182 mimic + let-7a mimic + NUAK1. **e** Protein levels of MMP2 and MMP9 detected by Western blot analysis upon treatment with miR NC, pc-DNA, miR-1182 mimic, let-7a mimic, miR-1182 mimic + let-7a mimic, miR-1182 mimic + let-7a mimic + NUAK1; ^*^ *p* < 0.05 vs. that of cells transfected with miR-NC or pc-DNA, ^#^ *p* < 0.05 vs. that of cells transfected with miR-1182 mimic or let-7a mimic, and & *p* < 0.05 *vs.* that of cells transfected miR-1182 mimic + let-7a mimic. The statistical data are measurement data and expressed as mean ± standard deviation. Comparisons among multiple groups were analyzed using ANOVA and followed by Tukey’s post hoc test. The experiment was repeated 3 times independently

To understand the role of miR-1182 and miR-1et-7a in progression of CAA, CCK-8 assay, Scratch test and transwell assay were carried out to detect the effects of miR-1182 and let-7a on HCCC-9810, Huh28 cell proliferation, migration and invasion. It was presented that, no significant difference in these capacities was observed in cells transfected with miR-NC and pc-DNA (*p* > 0.05). Compared with miR-NC and pc-DNA treatment, miR-1182 mimic, let-7a mimic and miR-1182 mimic + let-7a mimic weakened the capacities of migration, invasion and proliferation with treatment of miR-1182 mimic + let-7a mimic exhibiting inhibitory effect. But the presence of NUAK1 potentiated the cell progression, while simultaneously reversing the inhibitory effect of miR-1182 mimic and let-7a mimic (Fig. 5a–e, Additional file 2: Figure S1A, B).

Using RT-qPCR and Western blot analysis, the expression of MMP-2 and MMP-9 was determined, both of which are known to reflect migration [22] (Fig. 5f–g). There was no significant difference in cells transfected with miR-NC and pc-DNA (*p* > 0.05). The advent of either miR-1182 mimic or let-7a mimic declined the level of MMP-2 and MMP-9 in the cells, whilst combination of miR-1182 mimic and let-7a mimic resulted in a more remarkable inhibitory effect. However, the overexpression of NUAK1 in HCCC-9810 and Huh28 cells resulted in elevated levels of MMP-2 and MMP-9; treatment with miR-1182 mimic + let-7a mimic + NUAK1 rarely altered the level of MMPs as the level was similar to that of miR-NC or pc-DNA. Therefore, up-regulation of miR-1182 and let-7a can suppress both the migration and invasion of CCA cells, which can be reversed by NUAK1 overexpression. This indicates that miR-1182 and let-7a delays CAA cell progression through inhibition of NUAK1.

### Up-regulated miR-1182 and let-7a induce autophagy of CCA cells

**Fig. 6:**
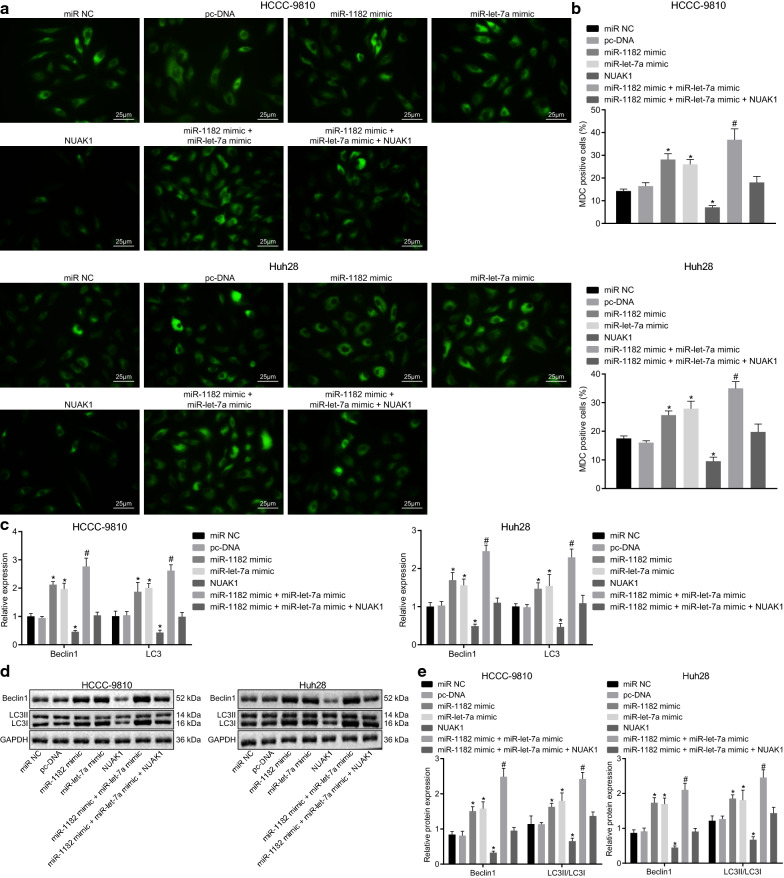
Up-regulated miR-1182 and let-7a induces cell autophagy. **a** MDC staining results (×400). **b** Statistical results of cell autophagy rate in each group. **c** The mRNA expression of Beclin1 and LC3 in HCCC-9810 and Huh28 cells measured by RT-qPCR. **d** The protein bands of Beclin1, LC3I, and LC3II determined by Western blot analysis. **e** Statistical results of the Beclin1, LC3I, and LC3II protein levels determined by Western blot analysis. ^*^ *p* < 0.05 vs. that of cells transfected with miR-NC or pc-DNA, ^#^*p* < 0.05 vs. that of cells transfected with miR-1182 mimic or let-7a mimic, and & *p* < 0.05 vs. that of cells transfected miR-1182 mimic + let-7a mimic. The statistical data were measurement data and expressed as mean ± standard deviation. Comparisons among multiple groups were analyzed using ANOVA and followed by Tukey’s post hoc test. The experiment was repeated 3 times independently

MDC staining was conducted to investigate the effect of miR-1182 and let-7a on cell autophagy. As results displayed, there was no significant difference in autophagy rate of cells transfected with miR-NC and pc-DNA (all *p* > 0.05). Compared with cells transfected with miR-NC and pc-DNA, autophagy rate was much higher in cells transfected with miR-1182 mimic, let-7a mimic and miR-1182 mimic + let-7a mimic. Combined treatment of miR-1182 mimic and let-7a mimic enhanced the promoting effect on autophagy. The autophagy fluorescence level was diminished in cells treated with NUAK1. When miR-1182 mimic + let-7a mimic + NUAK1 was treated simultaneously, the autophagy expression was restored to that level post miR-NC and pc-DNA (*p* > 0.05) (Fig. 6a, b).

Herein, we checked the expressions of autophagy related factors Beclin1 and LC3 using RT-qPCR and Western blot analysis upon treatment with overexpression of NUAKI and miRNAs. The levels of Beclin1 and LC3 mRNA were increased in cells transfected with miR-1182 mimic, let-7a mimic and miR-1182 mimic + let-7a mimic, with elevated ratio of LC3II/LC3I. Following treatment with NUAK1, the mRNA expression of Beclin1 and LC3 reduced dramatically, while LC3II/LC3I was also downregulated. The presence of oe-NUAK1 abrogated the effect of miR-1182 and let-7a mimic, as demonstrated by almost unchanged expression of Beclin1 and LC3 upon miR-1182 mimic + let-7a mimic + NUAK1 (Fig. 6c–e).

Collectively, overexpression of miR-1182 and let-7a promotes expression autophagy-related factor, enhancing autophagy while this result can be reversed by the up-regulation of NUAK1.

### Up-regulated miR-1182 and let-7a inhibit tumor growth in vivo

**Fig. 7:**
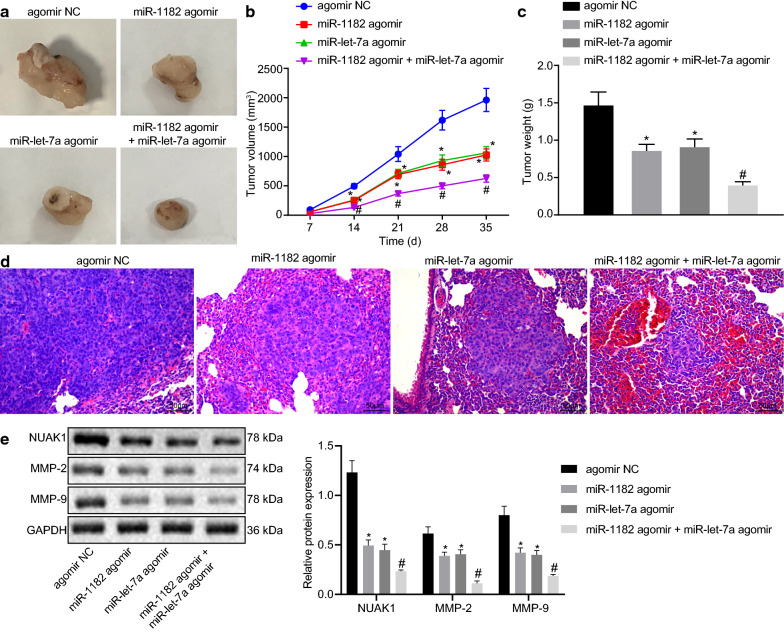
Up-regulated miR-1182 and let-7a prevent tumor growth in vivo. **a** Representative macroscopic picture of transplanted tumors upon treatment with agomir-NC, miR-1182 agomir, let-7a agomir, miR-1182 agomir + let-7a agomir. **b** The line graph of the tumor volume changes in nude mice. **c** Tumor weight of nude mice upon treatment with miR-1182 agomir, let-7a agomir, miR-1182 agomir + let-7a agomir. **d** Representative HE image (×200) of lung metastatic tumor upon treatment with agomir-NC, miR-1182 agomir, let-7a agomir, miR-1182 agomir + let-7a agomir. **e** Western blot analysis of NUAK1, MMP-2, and MMP-9 expression upon treatment with upon treatment with agomir-NC, miR-1182 agomir, let-7a agomir, miR-1182 agomir + let-7a agomir. *p* < 0.05 vs. treatment with agomir-NC, ^#^ *p* < 0.05 vs. treatment with miR-1182 agomir or let-7a agomir. The statistical data are measurement data and expressed as mean ± standard deviation. The tumor volume changes were compared with two-way ANOVA and followed by Bonferroni post hoc test

To understand the in vivo role of miR-1182 and miR-let-7 in tumor growth, the CCA animal models were established, followed by treatment with miR-1182 agomir, and miR-let-7 agomir. Upon treatment, we regularly measured the volume and weight of mice and found the tumor volume and weight of mice treated with miR-1182 agomir and let-7a agomir was much smaller than that of mice treated agomir-NC. The volume and the quality of the nude mice were markedly decreased in mice after miR-1182 agomir + let-7a agomir treatment (Fig. 7a–c). Results from HE staining revealed that CCA mice had clumps around the bronchus and bronchioles with lung metastasis where the alveolar structure was destroyed, and the distal lung metastasis tumor tissue had atelectasis. Upon treatment with overexpression of miR-1182 and let-7a, the symptoms were alleviated and only small focal pulmonary metastatic tumors were seen around the bronchus and bronchioles with a small number of necrotic cells. After the combined treatment of miR-1182 agomir + let-7a agomir, the capillaries and interstitial small blood vessels in the lung tissue of nude mice were dilated and congested, whilst lung metastasis was attenuated (Fig. 7d). Besides, Western blot analysis indicated that relative to agomir-NC, miR-1182 agomir and let-7a agomir decreased NUAK1, MMP-2, and MMP-9 expression and their combined treatment resulted in lower expression (Fig. 7e). The above results support that the application of miR-1182 in combination with let-7a down-regulates NUAK1 expression, resulting in the inhibition of tumor growth in nude mice.

## Discussion

CCA is the second most common primary hepatic malignancy, with an increasing incidence and mortality worldwide [23]. Early diagnosis is complicated due to the absence of specific symptoms during early stages of CCA, as a result of which most patients present at an advanced stage [24]. A thorough understanding of CCA shall therefore be prioritized. Our study revealed that miR-1182 and let-7a could play a role as tumor suppressors in CCA through the disruption of cell migration, invasion, proliferation, and autophagy by down-regulating NUAK1 expression. Furthermore, it has been previously proven that combination of miR-1182 and let-7a could have a synergistic inhibitory effect on CCA development.

One of the key findings from the present study showed that CCA tissues had a high expression of NUAK1. It has been suggested that NUAK1 was strongly associated with tumor invasion and migration, and also played an important role in tumor survival and progression [15]. Studies conducted on HCC have shown that high expression of NUAK1 has a positive regulatory effect on tumor size, histological differentiation, and TNM stage in HCC patients [25]; NUAK1 has also been identified as a promoter of invasion and migration in HCC cells through the regulation of epithelial mesenchymal transition [26]. Moreover, miR-145-regulated knockdown of NUAK1 reportedly leads to the inhibition of ICC cell proliferation, growth, and invasion, which contributed to clinical diagnosis and targeted therapy of ICC [16].

MiRNAs, such as hsa-miR-483-5p, hsa-miR-675, hsa-miR-139-3p, hsa-miR-598, hsa-miR-625 and hsa-miR-187, have a noteworthy involvement in cancer progression, due to their prognostic and predictive roles [27, 28]. In this study, CCA samples were observed to have low expressions of miR-1182 and let-7a. These two miRNAs have the potential to regulate NUAK1, in addition to their inhibitory action on cellular process. Both let-7a and miR-1182 have also been shown to be involved in tumorigenesis. Specifically, cervical cancer (CC) cells and tissues have significantly reduced let-7a expression, which promoted CC cell proliferation, migration and invasion by directly binding to 3′-untraslated region (UTRs) of pyruvate kinase muscle isozyme M2 (PKM2) [29]. Similarly, N Namwat et al. also revealed decreased let-7a expression in CCA samples [30]. Likewise, down-regulation of LINC00339, inhibited HCC cell growth, which in turn increased miR-1182 expression [31]. Additionally, other microRNAs, like miR-204, were found to have negative correlation with the expression of NUAK1, and miR-204 can act as a tumor suppressor in NSCLC tumor invasion through the down-regulation of NUAK1 [32]. Based on these results, it can be concluded that miR-1182 and let-7a have tumor suppressive effects on CCA via NUAK1 suppression.

Our study also revealed that elevated miR-1182 and let-7a down-regulated NUAK1 expression, resulted in the further inhibition of cell migration, invasion, proliferation as well as tumor growth and promoted autophagy. Several studies have looked into the relationship between miR-1182/let-7a and cell migration, invasion and autophagy. As an example, overexpressed miR-1182 could suppress telomerase reverse transcriptase (hTERT) expression and proliferation and invasion in gastric cancer (GC) cells [33]. In a study focused on bladder cancer study, overexpressed miR-1182 inhibited proliferation, colony formation, and invasion of cancer cell [34]. Similar results related to let-7a were also reported in GC, breast cancer and cholesteatomas. Specifically, the overexpression of let-7a significantly suppressed the proliferation, migration, and invasion of GC cells by down-regulating PKM2 expression [35]. Other studies have highlighted the key contribution of let-7a in breast cancer cell and cholesteatoma keratinocytes: let-7a suppressed migration and invasion of breast cancer cell by downregulating CCR7 expression [36], while it acts as a suppressor of growth and invasion of cholesteatoma keratinocytes [37]. The overexpression of let-7a was examined in LPS-stimulated BV2 microglial cells, the results of which found that it promoted the induction of the autophagy-related proteins such as LC3II, Beclin1. These results evidently demonstrated its potential role in the autophagy process [38]. Consistent with the previous example, let-7a increases cellular autophagic level through the inhibition of Rictor expression in GC cells [39]. The autophagy-related proteins include LC3, Beclin 1, and p62; LC3 serves as a specific marker of autophagosome formation and Beclin 1 has been identified as an essential modifier of the autophagic process, which is also involved in tumor development [40]. In this study, the expressions of Beclin1 and LC3II/LC3I were higher following the overexpression of miR-1182 and let-7a; however, this finding can be reversed by the overexpression of NUAK1, suggesting that the up-regulation of miR-1182 and let-7a could improve CCA cell autophagy.

## Conclusion

**Fig. 8:**
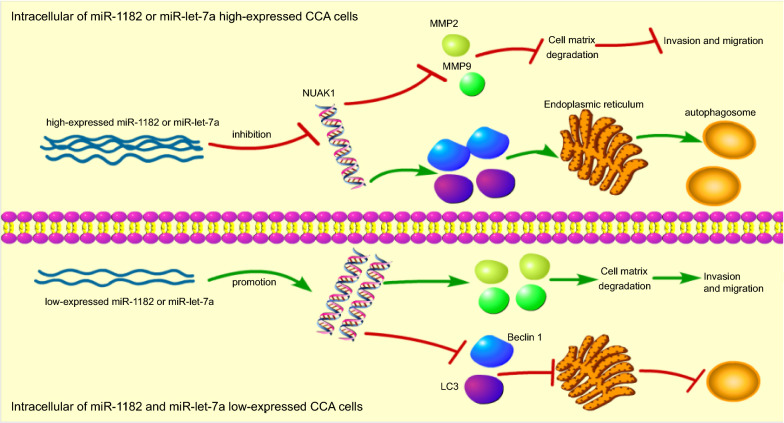
The effect of miR-1182 and let-7a on the development of CCA. In CCA, miR-1182 and let-7a are poorly-expressed, and NUAK1 gene is highly-expressed. MiR-1182 and let-7a regulate the expression of NUAK1 negatively. Overexpressed NUAK1 increases the levels of MMP-2/MMP-9, while represses the level of Beclin1, as well as LC3II/LC3I, with the results that cell migration, invasion are promoted and autophagy inhibited. MMP-2, matrix metallopeptidase 2; LC3I, light chain 3 I

In conclusion, miR-1182 and let-7a could facilitate a novel aspect in the treatment of CCA patients, on which they were found to exert synergistic inhibitory effects on cell migration, invasion, proliferation, and a promoting effect on cell autophagy through the down-regulation of NUAK1 expression (Fig. 8). Nevertheless, the limitations in the current study involved the lack of report on the potential role of miR-1182 or let-7a in CCA progression, which shall be explored in further studies.

## Supplementary Information

**Additional file 1: Table S1.** STR profiling of CCC-5, HCCC-9810 and Huh28 cell lines

**Additional file 2: Figure S1.** Up-regulated miR-1182 and let-7a disrupt cell invasion and migration. A, Representative images of cell migration distance at 0 h and 24 h using scratch test upon treatment with miR NC, pc-DNA, miR-1182 mimic, let-7a mimic, miR-1182 mimic + let-7a mimic, miR-1182 mimic + let-7a mimic + NUAK1 (scale bar = 250 um). B, Representative images of cell invasion images using transwell assay upon treatment with miR NC, pc-DNA, miR-1182 mimic, let-7a mimic, miR-1182 mimic + let-7a mimic, miR-1182 mimic + let-7a mimic + NUAK1 (scale bar = 50 um).

## Data Availability

The datasets generated/analysed during the current study are available.

## Abbreviations

CCA: Cholangiocarcinoma
miRNAs: MicroRNAs
ICC: Intrahepatic cholangiocarcinoma
NSCLC: Non-small cell lung cancer
MMP: Matrix metalloproteinase
DEGs: Differentially expressed genes
GEO: Gene Expression Omnibus
TCGA: The Cancer Genome Atlas
TNM: Tumor nodes metastasis
STR: Short tandem repeat
DMEM: Dulbecco’s modified eagle medium
RT-qPCR: Reverse transcription quantitative polymerase chain reaction
WT: Wild type
MUT: Mutated
FBS: Fetal bovine serum
NC: Negative control
oe: Overexpression
SDS-PAGE: Sodium dodecyl sulfate-polyacrylamide gel electrophoresis
HRP: Horseradish peroxidase
IgG: Immunoglobulin G
RIP: RNA Binding Protein Immunoprecipitation
RISC: RNA-induced silencing complex
CCK-8: Cell counting kit-8
MDC: Monodansylcadaverine
IHC: Immunohistochemistry
UTRs: Untranslated region
PKM2: Pyruvate kinase muscle isozyme M2
hTERT: Telomerase reverse transcriptase
GC: Gastric cancer
