# Supplementary material for: MicroRNA-1182 and let-7a exert synergistic inhibition on invasion, migration and autophagy of cholangiocarcinoma cells through down-regulation of NUAK1
Source: Cancer Cell Int. 2021 Mar 9;21:161. doi: 10.1186/s12935-021-01797-z (PMC7942015; doi:10.1186/s12935-021-01797-z)
Supplement: Supplementary file 1 — Additional file 1: Table S1. STR profiling of CCC-5, HCCC-9810 and Huh28 cell lines [file 12935_2021_1797_MOESM1_ESM.docx]

**Table S1** STR profiling of CCC-5, HCCC-9810 and Huh28 cell lines

|  | AMEL | D5S818 | D13S317 | D7S820 | D16S539 | vWA | TH01 | TPOX | CSF1PO |
| --- | --- | --- | --- | --- | --- | --- | --- | --- | --- |
| CCC-5 | X | 11,13 | 12 | 10,11 | 11,12 | 16 | 6,9.3 | 8 | 10,11 |
| HCCC-9810 | X | 11 | 11 | 10,12 | 9,12 | 16,17 | 9 | 8,11 | 11,12 |
| Huh28 | X | 9,12 | 9,12 | 10,11 | 9 | 17 | 9 | 8 | 9,12 |
